# A deep-learning algorithm (AIFORIA) for classification of hematopoietic cells in bone marrow aspirate smears based on nine cell classes—a feasible approach for routine screening?

**DOI:** 10.1007/s12308-025-00625-x

**Published:** 2025-03-29

**Authors:** Leonie Saft, Emma Vaara, Elin Ljung, Anna Kwiecinska, Darshan Kumar, Botond Timar

**Affiliations:** 1 Clinical Pathology and Cancer Diagnostics, Karolinska University Hospital, Stockholm, Sweden; 2 Department of Oncology-Pathology, Karolinska Institutet, Stockholm, Sweden; 3 Aiforia Technologies Plc, Helsinki, Finland; 4 1st Department of Pathology and Experimental Cancer Research, Semmelweis University, Budapest, Hungary

**Keywords:** Deep-learning algorithm, Hematopoietic cells, Bone marrow

## Abstract

Bone marrow cytology plays a key role for the diagnosis and classification of hematological disease and is often the first step in the acute setting of unclear cytopenia. AI applications represent a powerful tool in digital image analysis and can improve the diagnostic workflow and accuracy. The aim of this study was to develop an algorithm for the automated detection and classification of hematopoietic cells in digitized bone marrow aspirate smears for potential implementation in the clinical laboratory. The AIFORIA create platform (Aiforia Technologies, Plc, Helsinki, Finland) was used to develop a convolutional neural network algorithm based on nine cell classes. Digitized bone marrow aspirate smears from normal hospital controls were used for AI training. External validation was performed on separate data sets. Automated cell classification was assessed in whole-slide images (WSI) and regions of interest (ROI). A total of 1950 single-cell annotations were applied for AI training with a final total class error of 0.15% with 99.9% precision and sensitivity (FI-score 99.2%). External validation showed an overall precision and sensitivity of 96% and 97% and a F1-score of 96%. Automated cell classification correlated highly across ROI with variable correlation to WSI. The average execution time for classifying 500 hematopoietic cells was < 1 s and ≤ 260 s for WSI. A cloud-based, deep-learning algorithm for automated detection and classification of hematopoietic cells in bone marrow aspirate smears is a very useful, reliable, and rapid screening tool in combination with cytomorphology.

**Supplementary Information:**

The online version contains supplementary material available at 10.1007/s12308-025-00625-x.

## Introduction

The cytomorphological assessment of bone marrow aspirate (BMA) smears and/or imprints plays a central role in the diagnostic work-up of hematologic disease. It is often the first diagnostic test in the acute clinical setting of unclear cytopenia and suspicious leukemia and a highly effective screening tool if used in conjunction with flow cytometric immunophenotyping [1]. Apart from the assessment of cytomorphological details, BMA smears are used for differential cell counts (DCC) which can provide important diagnostic clues pointing to a broad range of benign and neoplastic hematologic disorders. The DCC is particularly critical in a subset of myeloid neoplasms where the defining diagnostic criteria specify percentage cutoffs for myeloid or other progenitor cells, e.g., in acute myeloid leukemia (AML) subtypes lacking recurrent genetic abnormalities, in myelodysplastic syndromes (MDS) and chronic myelomonocytic leukemia subtypes, or for establishing blast phase of myeloproliferative neoplasms [2, 3].

Previously published guidelines suggest that the DCC should be based on at least 500 cell count and comprise blast cells and the different maturation stages within granulopoiesis, promonocytes, monocytes, mast cells, lymphocytes, plasma cells, and erythroblasts [4]. However, the precise percentage of all cell types and maturation stages is not essential to the diagnosis in every case, such as samples taken as part of a routine BM staging examination.

The manual assessment of BMA smears is still considered the gold standard for DCC, but it is labor-intensive, time-consuming, and subject to inter- and intraobserver variability, emerging from the diversity and delicate intra-lineage difference within the maturation process of hematopoietic cells [5]. Digital pathology imaging coupled with deep-learning algorithms is a highly promising technology for this purpose. However, the automated detection and classification of hematopoietic cells in BMA smears are very challenging due to the high complexity of different cell morphologies, clustering and overlapping of cells, particularly in highly cellular smears, uneven distribution, and the presence of cellular artefacts.

Herein, we report our experience with the development of a deep-learning algorithm to detect and classify hematopoietic cells in BMA smears based on nine cell classes for use in the routine staging and screening examination. A brief section on recent studies on automated cell detection and classification applied to digital bone marrow images is included.

## Materials and methods

Bone marrow aspirate smears were scanned (Pannoramic 1000, 3DHISTECH Ltd, Budapest, Hungary) and uploaded to the AIFORIA create platform (Aiforia Technologies, Plc, Helsinki, Finland) for the development of a convolutional neural network (CNN)-based algorithm for the detection and classification of hematopoietic cells. External human validation was independently performed by three experts in bone marrow cytology on a separate set of digitized bone marrow images.

### Bone marrow aspirate smears

May-Grünwald Giemsa (MGG)-stained BMA smears were used for the training (*n* = 30), testing (*n* = 20), and validation (*n* = 30) of the AI model without duplication across datasets. The samples were collected from the archives of the Department of Clinical Pathology and Cancer Diagnostics, Karolinska University Laboratory (KUL), Solna. All samples were from untreated, non-cytopenic patients (*n* = 80) with normal or reactive marrow findings and taken as part of a routine bone marrow (BM) staging examination. The BMA smears were uniformly prepared using the same staining protocol according to the manufacturers’ guidelines (Sigma-Aldrich), and all included cellular marrow particles with the presence of megakaryocytes. The slides were digitized using a Pannoramic 1000 whole-slide scanner (3DHISTECH Ltd, Budapest, Hungary) with an output resolution of 63.06 × (using 40 × objective with a 1.6 × camera adapter magnification) and an image resolution of 0.158309 μm in *X* and 0.158834 in *Y* plane.

### Cell classification and annotation

**Fig. 1:**
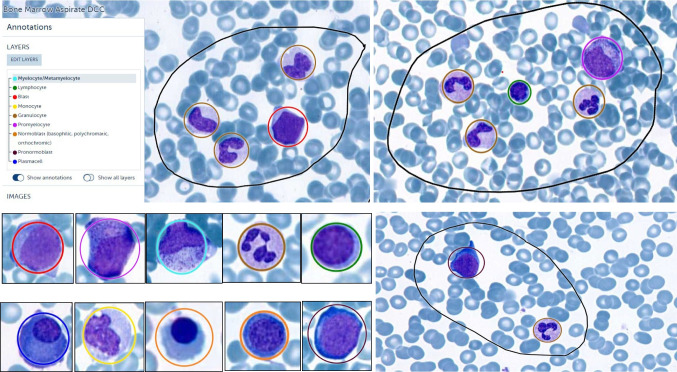
Training annotations based on nine cell classes in bone marrow aspirate smears. Single-cell annotations for nine cell classes: blast, *red ring*; promyelocyte, *purple ring*; myelocyte/metamyelocyte, *turquoise ring*; granulocyte (neutrophil, eosinophil, basophil), *brown ring*; lymphocyte, *green ring*; monocyte, *yellow ring*; plasma cell, blue ring; normoblast (orthochromatic, polychromatic, basophilic), *orange ring*; pro-normoblast, *bourgogne red ring*. The training regions are indicated by a black line, and all cells within these areas were annotated, except smudge cells, thrombocyte aggregates, and artefacts

The hematopoietic cells in BMA smears were assigned to nine major cell classes: blast, promyelocyte, myelocyte/metamyelocyte, proerythroblast, erythroblast (basophilic, poly- and orthochromatic), mature granulocytes (segmented/band neutrophil, eosinophil, basophil), lymphocyte, monocyte, and plasma cell. Mature granulocytes were combined into one class, with only a few eosinophils and very few basophils present in our data set. The various maturation stages within erythropoiesis were divided into two classes—the more immature proerythroblasts and normoblasts. The BM DCC did not include mast cells, megakaryocytes, smudge cells, and mesenchymal stromal cells. Representative examples of the cell classes used for training of the AI model are illustrated in Fig. 1.

Regions of interest (ROI) for annotation were first selected and drawn manually where cells were mostly evenly distributed, cytologically intact, non-overlapping, and best representative for the spectrum of hematopoiesis. Individual cells were annotated based on well-established cytomorphological criteria for each cell type [6] using a consistent cell size for each class with the whole target (nucleus and surrounding cytoplasm) centered (Fig. 1, Table 1). Advanced parameters were used to allow for “object” overlap and object size differences. All annotations were reviewed for appropriateness of classification (“ground truth”) by two experienced hematopathologists. Cells of uncertain class, smudge cells, naked nuclei, and thrombocyte aggregates were not annotated but included in the training regions.

**Table 1:** Cell classes in the AI model and number of annotations used for training

Cell classes (*n* = 9)	Cell size (µm) used for annotation	Cell size, range (µm)	No of annotated cells/class (% of total, *n* = 1950)
Blast	16	14–18	109 (5.6%)
Promyelocyte	18	12–20	202 (10.4%)
Myelocyte/metamyelocyte	16	10–18	294 (15%)
Granulocyte (neutrophil, basophil, eosinophil)	14	12–15	436 (22.4%)
Lymphocyte	10	8–10	191 (9.8%)
Monocyte	16	15–22	147 (7.5%)
Plasmacell	16	14–20	124 (6.4%)
Pronormoblast	18	12–20	142 (7.2%)
Normoblast (basophilic, polychromatic, orthochromatic)	12	12–17 8–12	305 (15.6%)

### Training and verification

Cell annotations were performed in a stepwise process following the recommended workflow (AIFORIA), starting with a smaller number of annotations for each class, followed by repeated training to guide new annotations until the desired AI model performance was obtained. The selected layer complexity for the model was set to “extra complex.” Advanced training parameters included, for example, the setting of maximum object overlap and minimal object size difference, in our model set at 0.5 and 0.25, respectively (Suppl. Table 1). Maximum object overlap prevents the neural network from finding two overlapping objects and, for example, detecting one object twice. Image augmentation was used to add variability to the training data during the training, i.e., more training data was created from the actual annotations. These parameters included the scale (min/max variation of training regions), luminance (min/max variation in the brightness within the same and in different images), contrast (different colors in the target regions in different images), all three set at min/max of − 10 to 10), maximum image shear (set at 10), maximum white balance change (set at 5), white noise (noise and artefacts in the background of the image (set at 2)), and rotation angle (min/max rotation angle used in augmentation, set at − 180 to 180). A total of 3056 (out of 7000) iterations were executed on all training regions (1 h 46 min 36 s) with an overall training loss of 0.2258.

Verification of the AI model was performed on the training regions and on selected areas outside the training regions to assess the generalizability of classification. Verification results were sorted by error rate (high-to-low) and used for reviewing the results. Annotations were improved by identifying misclassified cells and by adding annotations that were missed. Smudge cells, naked nuclei, cells in mitosis, thrombocyte aggregates, and cells that were not clearly identifiable were not annotated but intentionally included in the training region. The training was repeated several times with adjustment of the training parameters, and the AI model was further refined by alteration of the “gain values” for certain cell classes, if the model did not recognize enough or “too many” of that class. A total of 1950 single-cell annotations were performed for the training (Table 1). The final total class error for all training regions was 0.15% with 99.9% precision and sensitivity (FI-score 99.2%). Visual inspection of the classification results on a separate slide set that was not used for training indicated good performance of the AI model.

### External validation of the AI model

The AI model was validated against three external human validators, all three experienced in bone marrow cytology, using a separate set of digitized whole-slide images (WSI) from normal hospital controls (*n* = 20). The validation regions were areas in which cells were well dispersed with good cytological details and low number of smudge (lysed) cells. The external validators used their own computer screens and had access to the AIFORIA Create platform. An average of 2048 cell annotations in 515 validation regions were independently performed on two separate occasions. Annotations made by the human expert were considered the “gold standard,” and classification results of the three external validators were averaged for comparison to AI (“AI *vs* human”) and also compared to each other (“human *vs* human”) with respect to the “ground truth” generated by the training and testing of the AI algorithm.

### WSI analysis vs automated classification in regions of interest

In clinical routine, areas of well-spread marrow cells with good cytological details and paucity of artefacts are selected for the cytomorphological assessment of BMA smears and for performing DCC [4]. However, representative areas are not always found in the cellular trails of the BMA smear behind particles. For example, groups of blast cells can sometimes be detected in the tail or at the edges of the microscopic slides. Therefore, deep-learning models should either be applied on WSI or be trained for selecting ROI that are both informative and reflect the spectrum of hematopoietic cells present. Alternatively, a semi-automated approach could be used with the selection of ROI by human experts following WSI analysis for visual control of the output data in non-hemodiluted areas that show good cytological details. To test the appropriateness of the latter approach, 16 normal BMA smears were selected for WSI analysis and compared to the classification results in one larger ROI of equal size for all 16 samples vs ten smaller, randomly selected ROI/slide. The reason for also including a smaller ROI was that it better reflects the routine clinical approach when performing manual DDCs at high power magnification in different areas of a bone marrow aspirate smear.

### Statistical analyses

The classification results of the external validation were exported from the Aiforia Create Platform for statistical analysis. Statistical analyses were performed using R Statistical Software version 4.3.3.

False positive (FP) refers to objects that were not annotated (external validator), but detected by AI, and false negative (FN) refers to objects that were annotated (external validator) but not detected by the AI model. The false positive error was calculated by FP/(FP + TN), the false negative error by FN/(TP + FN), and the total class error by (FP + FN) / *P* where *P* is the sum of (TP + TN + FP + FN). Precision is the percentage of the analysis findings that overlap with annotated objects, calculated by TP/(TP + FP). Sensitivity is the percentage of annotated objects that were found by the analysis, calculated by TP/(TP + FN). The results of the external validation (Table 2) were calculated using a two-step averaging process by first calculating the average FP %, FN %, total error %, precision %, sensitivity %, and F1-score for the nine cell classes per validator. The calculated values were then averaged across the three validators and compared to AI. The reported F1-score is the average of the F1-score from the three validators.

**Table 2:** External validation of the AI model (“AI vs human”) and comparison of classification results between experts

Method	Cell class	False positive error (%)	False negative error (%)	Total error (%)	Precisio*n* (%)	Sensitivity (%)	F1-score (%)
AI vs human	Blast	12.94	0.76	5.79	94.97	99.24	95.90
Human vs human		0.93	0.46	0.93	99.54	99.54	99.38
AI vs human	Promyelocyte	10.02	2.48	6.87	95.44	97.52	95.29
Human vs human		5.72	2.96	5.66	97.03	97.03	96.16
AI vs human	Myelocyte/metamyelocyte	16.18	9.45	17.04	91.05	90.55	88.56
Human vs human		16.30	8.74	16.51	91.26	91.26	88.93
AI vs human	Granulocyte	6.16	1.87	5.55	96.13	98.13	96.62
Human vs human		4.98	2.86	5.62	97.14	97.14	96.52
AI vs human	Lymphocyte	5.75	4.34	7.04	97.30	95.66	95.14
Human vs human		5.84	2.76	5.51	97.25	97.25	96.32
AI vs human	Plasmacell	1.05	0.35	1.05	99.30	99.65	99.35
Human vs human		0.34	0.17	0.34	99.83	99.83	99.77
AI vs human	Monocyte	7.01	3.20	6.66	96.37	96.80	95.50
Human vs human		4.42	2.15	4.31	97.85	97.85	97.12
AI vs human	Pronormoblast	4.18	0.29	2.38	97.91	99.71	98.42
Human vs human		0.94	0.5	1.00	99.50	99.50	99.35
AI vs human	Normoblast	3.03	4.23	5.79	98.33	95.77	96.26
Human vs human		5.04	2.76	5.34	97.24	97.24	96.52

False positive (FP) error (%): objects that were not annotated (external validation), but detected by AI, calculated by FP/(FP + TN), where TN refers to true negatives

False negative (FN) error (%): objects that were annotated (external validation), but not detected by AI, calculated by FN/(TP + FN), where TP refers to true positives

Total class error (%): (FP + FN) / *P*, where *P* is the sum of (TP + TN + FP + FN)

Precision: percentage of the analysis findings that overlap with annotated objects, calculated by TP/(TP + FP)

Sensitivity: percentage of annotated objects that were found by the analysis, calculated by TP/(TP + FN)

F1-score (%): harmonic mean of precision and sensitivity

The Shapiro–Wilk test was used to assess whether the data sets from whole slide image (WSI) analysis and regions of interest (ROI) were normally distributed. The Spearman rank correlation test was used to assess the correlation between the classification results from WSI analysis and ROI.

## Results

### External human validation against the AI model

**Fig. 2:**
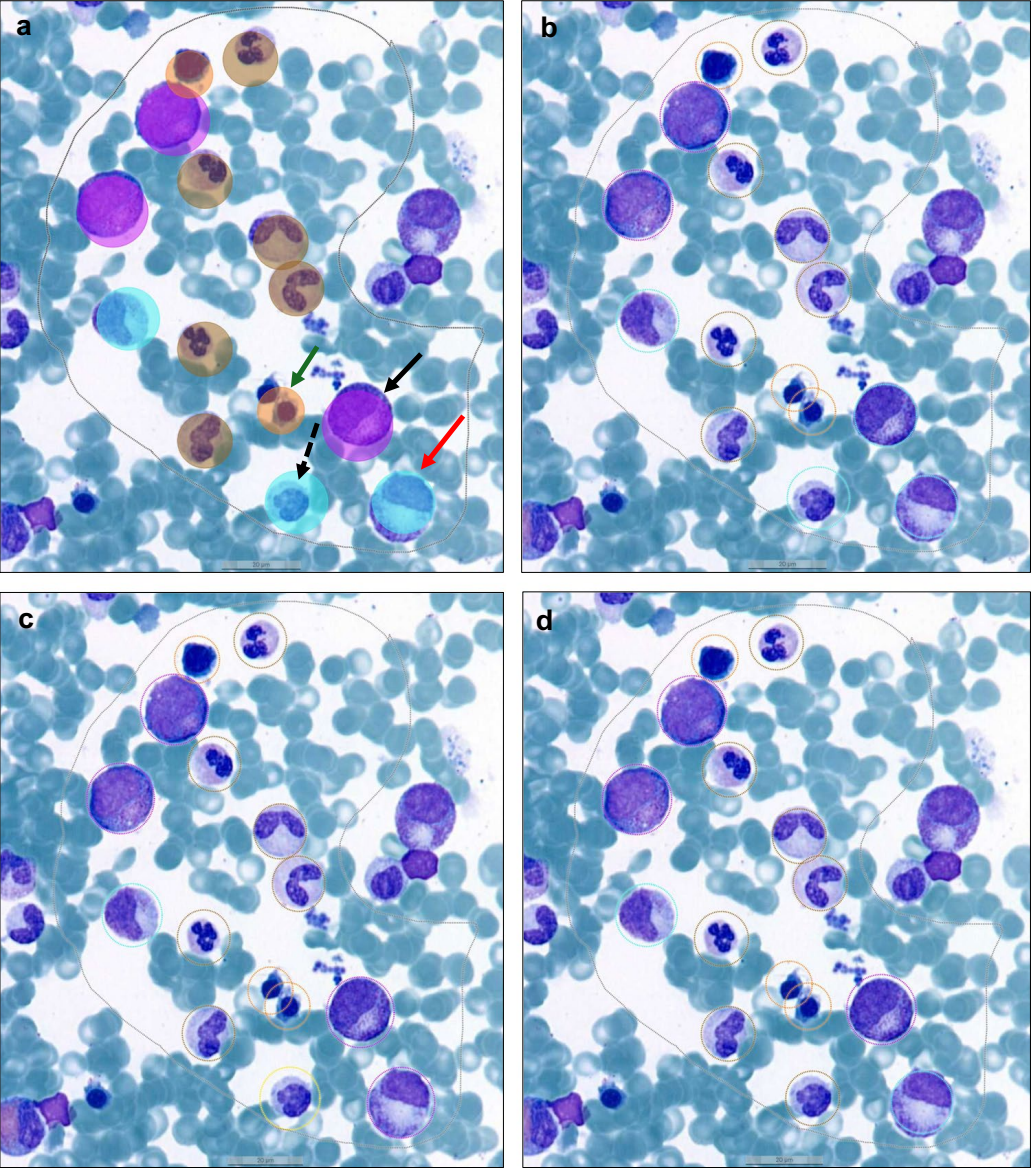

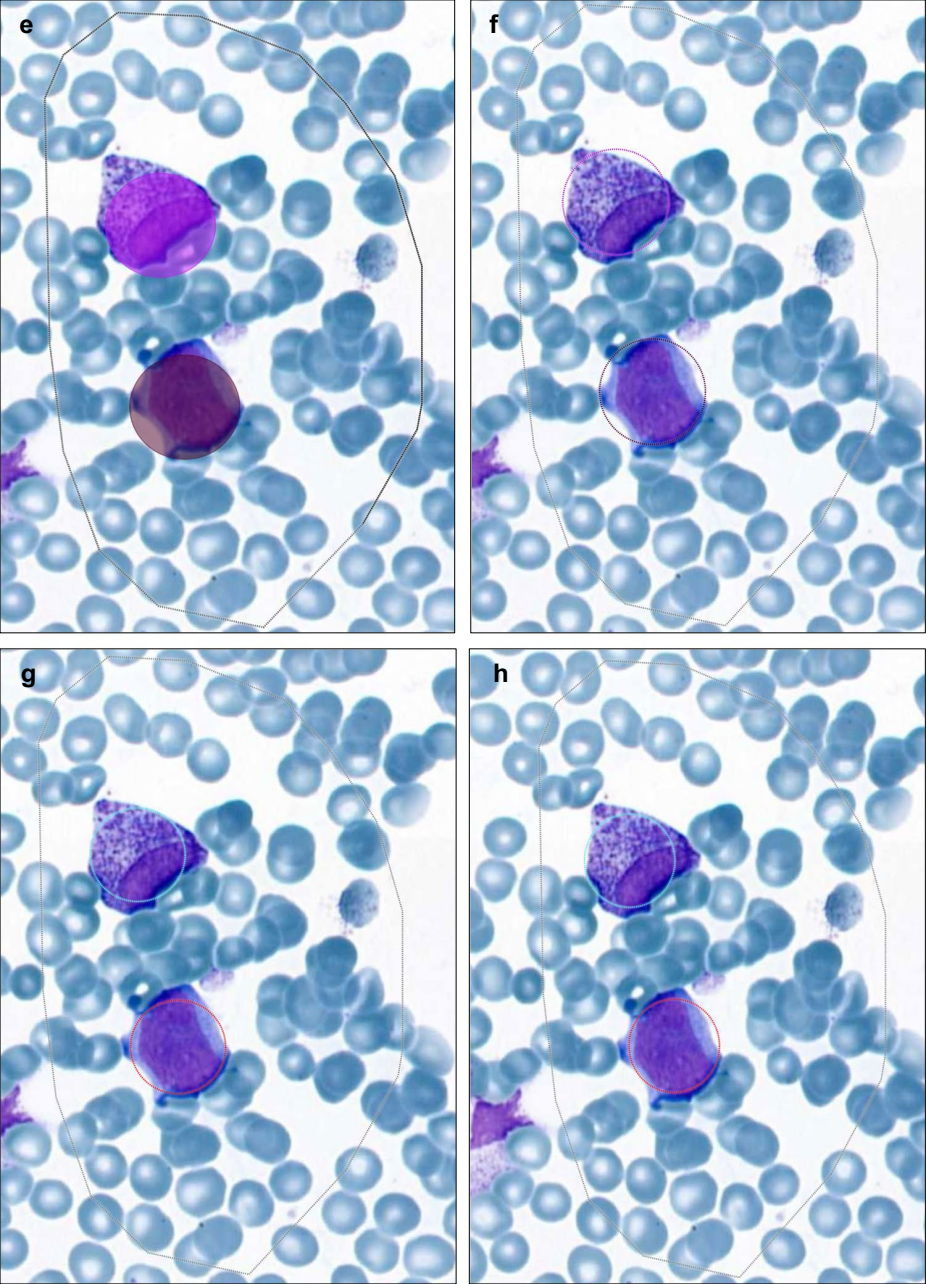
Single-cell annotations in validation regions performed by three human experts and comparison to the AI model. External human validation of the AI model. The different plots show the annotation results generated by the AI model (plots **a** and **e**, shaded cell) and the three external human validators (plots **b**–**d**, **f**–**h**, ring annotations). The different cell classes are annotated by colored rings (see Fig. 1 for detailed explanation) and discrepant results are depicted by arrows. Black arrow: annotated as promyelocyte by AI and two examiners (**c**, **d**) and as myelocyte by one examiner (**b**). Red arrow: AI and two examiners annotated this cell as myelocyte and as promyelocyte by the third examinator (**c**). Dotted black arrow: annotated as myelocyte by AI and B and as monocyte (**c**) and granulocyte (**d**) by the two other examiners. The second image (plots **e**–**h**) illustrates differences in the classification of blast cells (*red* ring) vs pronormoblasts (*bourgogne red* ring) and in the classification of promyelocytes (*purple* ring/shadowed cell) vs myelocyte/metamyelocyte (*turquoise* ring)

The results of the external validation by three human experts against the AI model and comparison between the external validators are summarized in Table 2; examples of classification results are illustrated in Fig. 2. The overall precision and sensitivity for “AI vs human” were 96% and 97%, respectively (F1-score 96%), and 97.4% for both when comparing “human vs human” (F1-score 96.67%). The error percentages are mean values obtained from the three combinations coming from the three external validators (human vs human) and the three individual comparisons of the AI model against human experts (AI vs human). The mean total object error for “AI vs human” and “human vs human” was 6.46% and 5%, respectively, and lowest for plasma cells (1.05% vs 0.34%) and pronormoblasts (2.38% vs 1%) for both comparisons.

The difference of false positive and false negative errors (“AI vs human” and “human vs human”) was small for the various classes, except for blasts, promyelocytes, and pronormoblasts with higher FP (%) for “AI vs human.” The error % for blasts refers to results generated below the critical 5% blast threshold, since the samples were from normal hospital controls without blast increase. A closer visual control of “misclassifications” illustrates difficulties in distinguishing blasts and pronormoblasts, but also promyelocytes and myelocytes for both comparisons (Fig. 2). This may, at least in part, be explained by differences in size and cell morphologies within cell classes in the same sample but also across samples used for training. Comparison to cells belonging to the same cell class outside validation regions provided guidance for correct classification by the human validator.

The false positive rate of classifying hematopoietic cells was highest for “myelocytes/metamyelocytes,” reflecting subtle morphological changes within different maturation stages in granulopoiesis. On closer visual inspection of cell classes that were misclassified by AI but correctly classified by all three validators, it became evident that the algorithm had difficulties in distinguishing between monocytes and metamyelocytes or band neutrophils. Specific classification errors and disagreement for both comparisons included, as stated above, different maturation stages within the myeloid and erythroid series (band neutrophil vs metamyelocyte; myelocyte vs promyelocyte; blast vs pronormoblast).

### WSI analysis vs automated classification in ROI

**Fig. 3:**
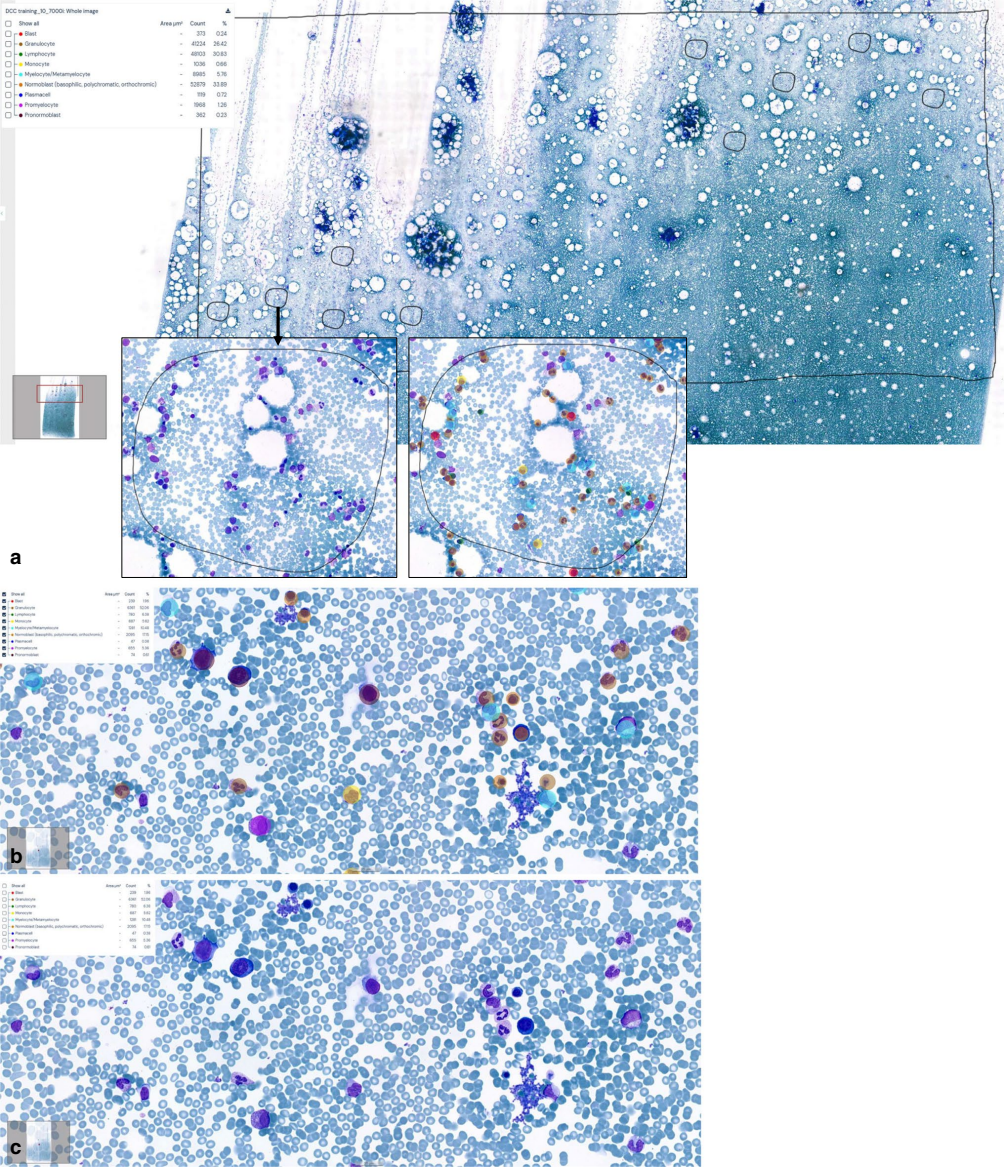
Automated detection and classification of hematopoietic cells in whole-slide images and regions of interest. **a** Example of WSI analysis with the results shown in the box (upper left corner); classification results were compared to one larger ROI (square) and several smaller ROI (circle). **b** and **c** WSI analysis with (“masked cells”) and without the classification results visually shown for one area of the slide at higher magnification; the total number of detected objects/cell class is shown in the box

**Fig. 4:**
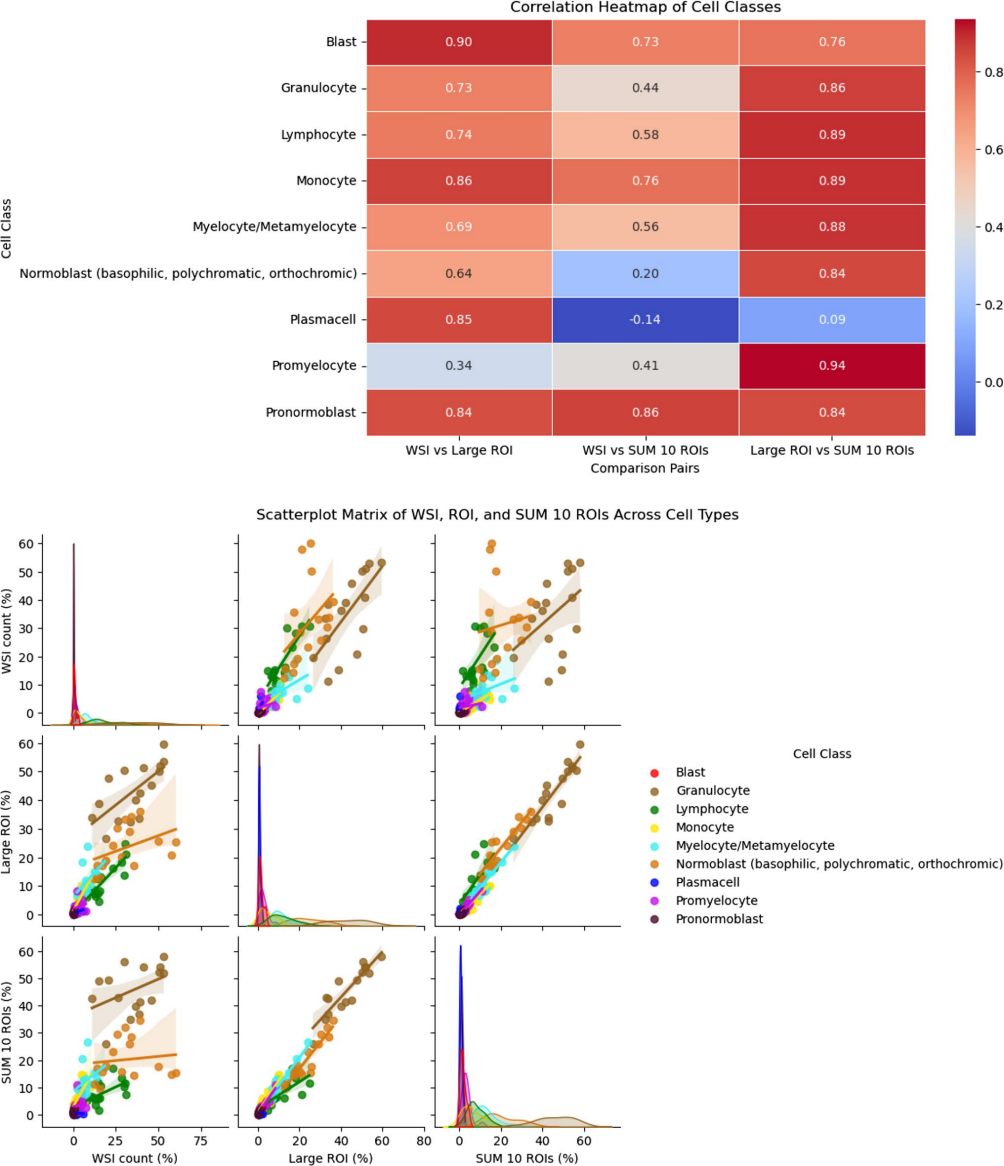
Spearman’s rank correlation heatmap and Pairwise Scatterplot Matrix for WSI analysis and ROI across nine cell classes. The heatmap visualizes Spearman’s rank correlation coefficients (*ρ*) for the nine cell classes in WSI compared to one larger ROI and ten smaller ROI. The color intensity reflects the strength of the correlation, with dark red indicating a strong positive correlation. The scatterplot matrix illustrates the correlation for the cell detection counts (%) in WSI, large ROI, and smaller ROI for nine cell classes. Each subplot represents a pairwise comparison with individual regression trend lines indicating correlation patterns. Different cell classes are color-coded to facilitate comparison. The diagonal plots display kernel density estimates (KDE) to illustrate the distribution of each variable within the dataset

The detailed detection and classification results for WSI analysis and ROI are provided in Suppl. Table 2. Figure 3 illustrates the approach showing one larger ROI and ten smaller ROI that were separately analyzed and compared to classification results of WSI analysis. The three datasets (WSI, larger ROI, ten smaller ROI/slide) showed a non-normal distribution (Shapiro–Wilk test). The Spearman rank correlation test was used to assess the correlation for the different cell classes in WSI analysis vs one large ROI vs ten smaller, randomly selected ROI. The datasets were grouped by cell classes and the test results were visualized using a heatmap (Fig. 4). WSI analysis and larger ROI correlated highly for several classes, including blasts. The presence of outliers in the dataset from WSI, particularly for normoblasts, indicates skewness. The visual review of the classification results for these outliers in the corresponding bone marrow smears shows larger areas with poorly preserved cellular details that do not allow reliable cell classification and hemodiluted areas. All 16 samples had higher lymphocyte counts in WSI compared to ROI.

All ROI were manually selected in areas of the bone marrow aspirate smear that showed adequate cellularity and well-preserved cytomorphological details with a paucity of artefacts. The classification results correlated highly for all cell classes when comparing “ROI large” with the sum of ten smaller ROI of the same slide, except for plasmacells that were present in very low numbers in the whole dataset and often not present in the smaller regions.

The total number of detected cells (counts) in WSI and larger ROI was ranging between 27,893 and 677,698 counts/slide and between 6864 and 75,044 counts/slide, respectively. The total counts in ten smaller ROI were ranging between 270 and 1303 cells/slide. The execution time was ≤ 1 s for detecting and classifying approximately 500 cells and between 129 and 260 s for WSI, dependent on the total cell count per slide.

### Previous studies on cell detection and classification in bone marrow aspirate smears

During recent years, a few studies have been devoted to the automatization of BMA DCCs in digital images. Choi et al. published promising results using a dual-stage convolutional network (CNN) for cell classification in BMA smears based on ten classes of the myeloid and erythroid maturation series and achieved a precision of 97.13% and a F-1 score of 97.1% [7]. The data set from Choi et al. was used for external validation in a recent study by Matek et al. [8]. In this single-center study, DNN was applied to > 170,000 expert-annotated microscopic images from 945 adult patients diagnosed with a large variety of hematological malignancies and reactive conditions, reflecting the sample entry of a single large laboratory specialized in hematology. ROI were manually selected by human experts for morphological analysis with cell annotation to 21 classes. The model achieved high accuracy and the external validation indicated that the method was generalizable to data obtained in other settings.

The technical approach and workflow used in our study were similar to the one described by Chandradevan et al. (2020) who developed a CNN-based model for automated DCC of non-neoplastic BMA smears based on 11 cell classes [9]. The samples were from one single center, scanned, and uploaded to a digital slide archive server for annotation in manually selected ROI. The average execution time for cell detection and classification was less than 3 min for ROI containing 500 cells.

Fu et al. developed the automatic CNN-based system Morphogo to classify and analyze nucleated cells in BMA smears using > 3000 archived BMA smears from patients with reactive and neoplastic conditions [10]. In their study, nucleated cells were assigned to 12 categories, including the different myeloid maturation stages and one erythroid class, with a reported classification accuracy of above 85.7%. The automated and manual classification results correlated highly with respect to granulocytes, erythroid precursors, and lymphocytes (*r* ≥ 0.762), but showed low or no correlation for monocytes (*r* < 0.459) and blasts.

A fully automatic hierarchical deep-learning framework for BMA DCC of WSI based on 16 cell classes was recently described by Wang et al. [11]. The reported accuracy was 0.989 and the computational time was 44 s for a WSI. The model differs from previous studies by its fully automatic approach on WSI without human intervention by manually selected ROI. As reported in the other studies, monocytes presented a challenge in the recognition task due to overlapping features with other cell types.

A newly published multicenter study presents a novel computational approach with an integrated AI decision support system (Scopio Labs X100 full Field BMA) that operates by a cloud-based application allowing a fully remote BM analysis and reporting without the requirement of specific software installation [12]. A comparative analysis based on 795 BMA samples, stained with different, site-specific protocols from patients with various diagnoses, was performed. The multi-center agreement between the test (AI model) and reference method (manual) for the BMA assessment was high, with 93.58% agreement for specimen quality and 84.03% for cell counts.

Another recent study proposed a system based on a three-dimensional (3D) printed device that couples a smartphone to a conventional optical microscope, allowing the acquisition of microscopic images [13]. The acquired images were transferred to a web-based telemedicine platform for automated cell classification. The proposed system could, in theory, be implemented at any workplace without incorporating complex medical electronic devices into the clinical workflow.

Most previous studies followed a single-center approach with BMA smears included for training prepared in the same laboratory and digitized using the same scanning equipment. Within that setting, the algorithms described showed encouraging performance with high classification accuracy. However, these studies also reflect common difficulties for developing such models due to the complexity of BM cytology and high intra-class differences in individual samples resulting from the continuous maturation process. One study performed external validation by using datasets from another center which indicated that their method was generalizable to data obtained in other settings [8].

## Discussion

Herein, we present a deep-learning algorithm for the detection and classification of hematopoietic cells in digitized BM images from normal hospital controls. Given the complexity of bone marrow cytology and the difficulties in developing reliable qualitative analytic tools on one hand and the widespread use of digital images in clinical pathology on the other hand, we attempted to develop a simple and quick AI model for use in the routine screening examination of bone marrow aspirates based on fewer (nine) cell classes as compared to previous studies. This approach is supported by the lack of clinically meaningful highly complex DCC in the majority of cases that enter the hematopathology laboratory. In the workup of unclear cytopenia and a suspected myeloid neoplasm, a full 500-cell DCC on the bone marrow aspirate smear, as recommended by the WHO and the International Council for Standardization in Hematology (ICSH), is usually warranted [4, 14]. In *Dacie and Lewis Practical Hematology*, it is stated that a 200- to 500-cell differential using the categories erythroid, myeloid, lymphoid, and plasma cells is generally adequate provided that a systematic scheme for examining the morphology is used [15]. One study suggested that a 300-cell DCC may be sufficient for most cases, even for evaluation of myeloid and plasma cell neoplasms [16].

We developed a reliable AI algorithm with high precision and accuracy by artificially generating and expanding the ground truth using AIFORIAS hyperparameters, yielding similar outputs as described in previous studies that were based on much higher numbers of images and annotations. The accuracy of the model was further improved by using the “human-in-the-loop” (HITL) approach in the review process of the AI model’s performance. Mori et al. 2020 developed an AI system for the prediction of dysplasia in BMA smears from patients with myelodysplastic syndrome (MDS) and used the HITL strategy to correct misclassifications by both the AI system and human examiners [17]. In the routine clinical setting, the “human-in-the-loop” principle may offer advantages where both morphologists and deep-learning algorithms fall short, e.g., by using rapid automatic cell detection and visual control of the classification results by the human expert with the integration of the qualitative morphological assessment and clinical data [18].

Importantly, the automated approach can never replace the cytomorphological assessment and a thorough review of the bone marrow aspirate smear in the clinical context, and correlation with other morphologic and ancillary data is still a necessary and standard approach taken by hematopathologists. In this setting, the authors favor a semi-automatic approach based on the manual selection of representative, preferably larger ROI of good quality for visual control and comparison to the classification results in WSI. This is supported by our data with a high correlation for classification results when comparing larger with several smaller ROI in well-preserved areas of the BMA smear.

In this study, we only included a limited number of non-neoplastic BMA smears from adult patients in the training and the full spectrum of reactive conditions was certainly not represented. Furthermore, we employed a relatively small ROI in the training and validation sets, biased towards better cytologic preservation, which is a limitation, but which also reflects the approach used in clinical routine. Another important aspect is the assessment of blast percentage with respect to critical thresholds according to current classifications of myeloid neoplasms (WHO/ICC) using AI techniques. It is well-known that blast enumeration is subject to sampling variations/error and subjective evaluation, and a single gold standard for blast enumeration does not exist. The samples used in this study were from normal hospital controls (all had < 5% total marrow blasts) with a relatively high false positive rate for AI below the critical 5% threshold, indicating difficulties in distinguishing blasts and pronormoblasts, while the false negative rate was very low. This may be due to variations in cell morphologies and blast size within samples, but also across different samples that were used for training of the AI model. Comparison to cell morphologies outside validation regions provided guidance for correct classification made by the three human experts. Therefore, larger training regions could improve AI performance for the correct classification of blast cells. We are planning to perform additional studies using BMA smears and bone marrow biopsies in parallel, including neoplastic samples with various blast percentages, combined with other sensitive techniques (e.g., flow cytometry).

Digital imaging technology coupled with deep-learning algorithms represents a rapidly emerging technology for automating DCCs. Aside from reducing labor costs, such approaches could potentially improve accuracy, reproducibility, and objectivity and provide standardization for DDCs. Although the implementation of AI algorithms in daily clinical practice is imminent, the applicability is still hampered by domain divergence (different scanners, stainers, antibodies). Large-scale multicenter studies on routine hospital samples including a range of scanner hardware to increase the performance and robustness of future algorithms are needed for validation and potential implementation in the clinical laboratory. Various browser-based solution systems, as presented in a very recent study [12], may represent excellent evaluation tools for fully remote BMA analysis and reporting and for use as external quality assessment and training programs. As a next step, we are aiming at testing the proposed algorithm in a wider range of reactive conditions and neoplastic hematological diseases engaging the bone marrow in both children and adults. The integration of data from several examinations (e.g., BM biopsy, flow cytometry, genetics) to construct a multimodal deep-learning network and to improve the integrity of the procedure will be important tasks in the future.

## Supplementary Information

Below is the link to the electronic supplementary material.

Supplementary file1 (DOCX 17 KB)

Supplementary file2 (PPTX 4695 KB)

Supplementary file3 (DOCX 43 KB)

## Data Availability

No datasets were generated or analysed during the current study.
