# Supplementary material for: A deep-learning algorithm (AIFORIA) for classification of hematopoietic cells in bone marrow aspirate smears based on nine cell classes—a feasible approach for routine screening?
Source: J Hematop. 2025 Mar 29;18(1):12. doi: 10.1007/s12308-025-00625-x (PMC11954740; doi:10.1007/s12308-025-00625-x)
Supplement: Supplementary file 1 — Supplementary file1 (DOCX 17 KB) [file 12308_2025_625_MOESM1_ESM.docx]

**Suppl. Table 1**. Advanced parameters and image augmentation used for the training of the AI model.

| **Advanced parameter** | **Value** | **Image augmentation** | **Value** |
| --- | --- | --- | --- |
| Complexity | Extra complex | Scale (min/max) | -10 to 10 |
| Weight decay | 0.0001 | Aspect ratio | 10 |
| Mini-batches per iteration | 20 | Maximum shear | 10 |
| Initial learning rate | 1 | Luminance & contrast (min/max) | -10 to 10 |
| Window size | 200 px | Maximum white balance change | 5 |
| Maximum object overlap | 0.5 | Noise | 2 |
| Minimum object size difference | 0.25 | Rotation angle (min/max) | -180 to 180 |

**Table explanations:**

**Advanced parameters** are detailed settings for training and image analysis. Weight decay is used to improve model performance by penalizing larger weights in the network to prevent overfitting. Mini-batches split the training data set into small batches to avoid running out of memory in large data sets; a larger value slows down iterations but speeds up the training. Initial learning rate controls how quickly or slowly a neural network model learns a feature. The window size depends on the sizes of the objects. Maximum object overlap prevents the neural network finding two overlapping objects, and detecting one object twice. This feature is useful when cells are overlapping, as frequently seen in BMA smears. Minimum object size difference controls the granularity of the detector windows.

**Image augmentation** means that the system adds variability to the training data during the training, i.e. more training data is created from the actual annotations. Augmentation can help preventing the AI model to over-learn specific image details, and focus on essential patterns.

Scale is used when the regions vary in size. Aspect ratio is used for regions and objects of, for example, variable thickness or here cellulular levels. Maximum shear refers to the changing the shape and size of a 2D object along X- and Y-axis. Maximum white balance change is used as a reference for the white balance change percentage. Noise is added to the training images which helps to teach the neural network to ignore noise and artefacts. The minimum and maximum image rotation angles are used in augmentation.
