# Supplementary figures and images for: A deep-learning algorithm (AIFORIA) for classification of hematopoietic cells in bone marrow aspirate smears based on nine cell classes—a feasible approach for routine screening?
Source: J Hematop. 2025 Mar 29;18(1):12. doi: 10.1007/s12308-025-00625-x (PMC11954740; doi:10.1007/s12308-025-00625-x)

## Slide 1
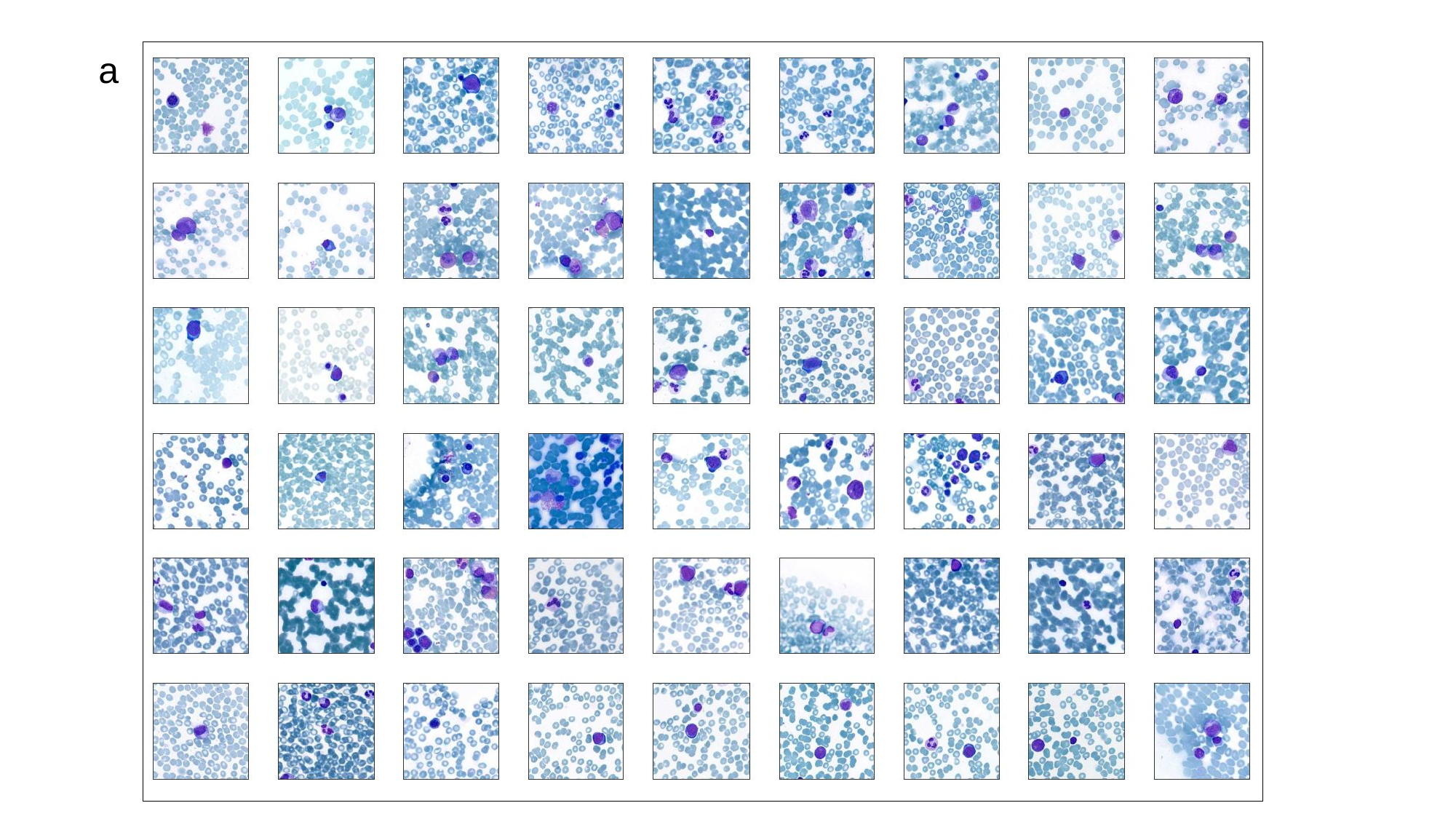

a

## Slide 2
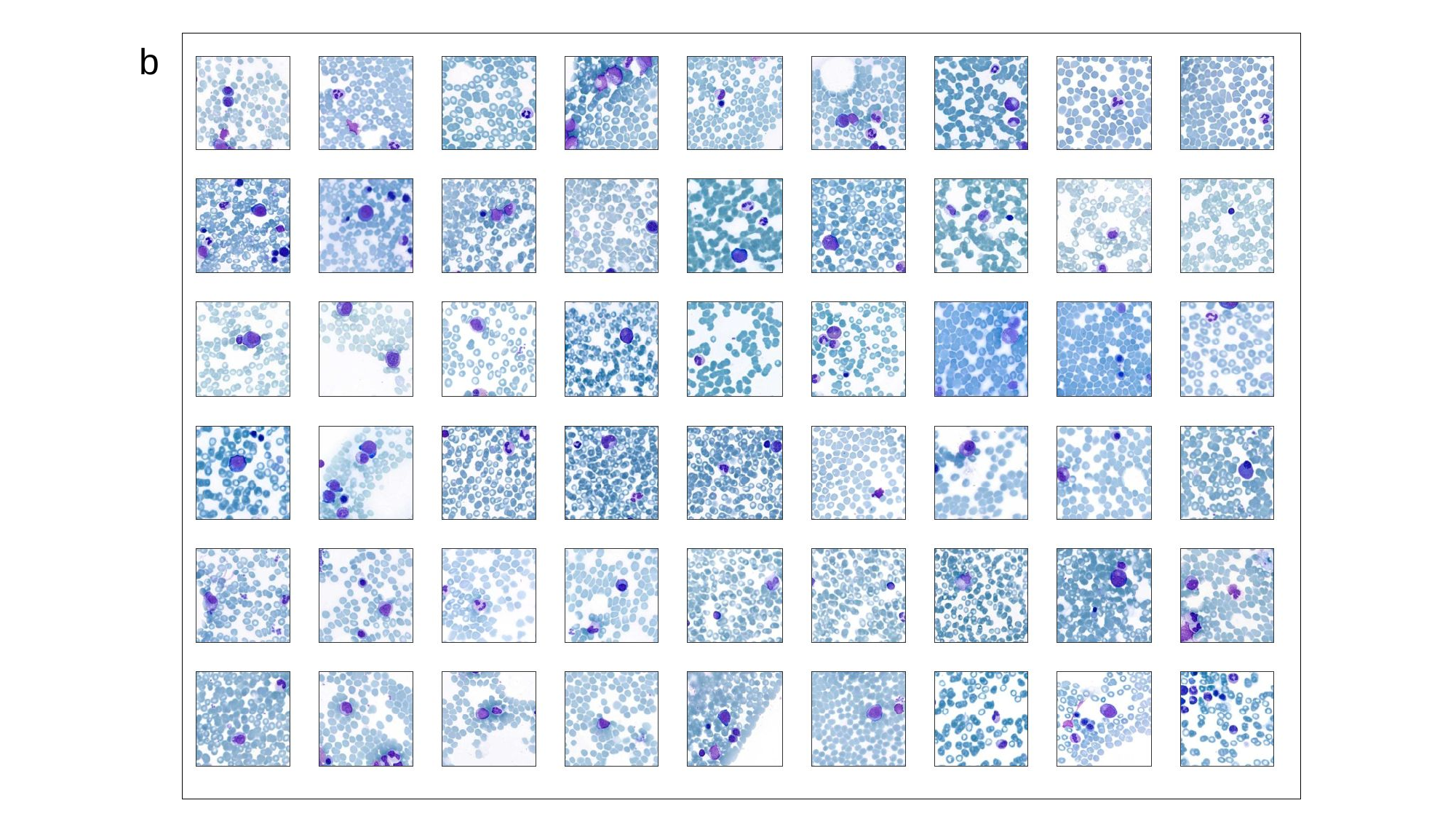

b

Supplement: Supplementary file 2 — Supplementary file2 (PPTX 4695 KB) [file 12308_2025_625_MOESM2_ESM.pptx]
