# Supplementary material for: A deep-learning algorithm (AIFORIA) for classification of hematopoietic cells in bone marrow aspirate smears based on nine cell classes—a feasible approach for routine screening?
Source: J Hematop. 2025 Mar 29;18(1):12. doi: 10.1007/s12308-025-00625-x (PMC11954740; doi:10.1007/s12308-025-00625-x)
Supplement: Supplementary file 3 — Supplementary file3 (DOCX 43 KB) [file 12308_2025_625_MOESM3_ESM.docx]

| **Suppl. Table 2. WSI analysis of bone marrow aspirate smears and comparison to classification results in ROI** | | | | |  |  |  |
| --- | --- | --- | --- | --- | --- | --- | --- |
|  |  |  |  |  |  |  |  |
| **Slide** | **Cell Class** | **WSI, total count** | **Large ROI, total count** | **Smaller ROI (n= 10), total count** | **WSI count (%)** | **Large ROI (%)** | **Smaller ROIs (n=10) (%)** |
| **1** | Blast | 521 | 239 | 7 | 0.975290154 | 1.95597021 | 1.741293532 |
|  | Granulocyte | 27312 | 6361 | 218 | 51.12691876 | 52.05826991 | 54.22885572 |
|  | Lymphocyte | 6953 | 780 | 14 | 13.01572445 | 6.383501105 | 3.482587065 |
|  | Monocyte | 1885 | 687 | 28 | 3.528640958 | 5.622391358 | 6.965174129 |
|  | Myelocyte/Metamyelocyte | 4280 | 1281 | 45 | 8.011980532 | 10.48367297 | 11.19402985 |
|  | Normoblast | 9271 | 2095 | 63 | 17.35492325 | 17.14542925 | 15.67164179 |
|  | Plasmacell | 136 | 47 | 1 | 0.254586297 | 0.384646861 | 0.248756219 |
|  | Promyelocyte | 2890 | 655 | 19 | 5.409958817 | 5.360504133 | 4.726368159 |
|  | Pronormoblast | 172 | 74 | 7 | 0.321976788 | 0.605614207 | 1.741293532 |
| **2** | Blast | 1684 | 672 | 23 | 2.616125524 | 3.79039991 | 4.197080292 |
|  | Granulocyte | 21503 | 5766 | 191 | 33.40531303 | 32.52298494 | 34.8540146 |
|  | Lymphocyte | 8149 | 1286 | 32 | 12.65962405 | 7.253652208 | 5.839416058 |
|  | Monocyte | 4132 | 1055 | 25 | 6.419139351 | 5.950702239 | 4.562043796 |
|  | Myelocyte/Metamyelocyte | 8184 | 2645 | 85 | 12.7139972 | 14.91905917 | 15.51094891 |
|  | Normoblast | 16582 | 5359 | 162 | 25.76044741 | 30.22731119 | 29.5620438 |
|  | Plasmacell | 302 | 65 | 0 | 0.469162653 | 0.366630944 | 0 |
|  | Promyelocyte | 3271 | 653 | 20 | 5.081559733 | 3.683230865 | 3.649635036 |
|  | Pronormoblast | 563 | 228 | 10 | 0.874631039 | 1.286028541 | 1.824817518 |
| **3** | Blast | 499 | 111 | 2 | 0.446328745 | 1.116924935 | 0.626959248 |
|  | Granulocyte | 45830 | 5097 | 173 | 40.99247771 | 51.28798551 | 54.23197492 |
|  | Lymphocyte | 26109 | 1617 | 54 | 23.3531006 | 16.27087945 | 16.92789969 |
|  | Monocyte | 1001 | 218 | 6 | 0.895340829 | 2.193600322 | 1.880877743 |
|  | Myelocyte/Metamyelocyte | 4821 | 681 | 19 | 4.31212601 | 6.85248541 | 5.956112853 |
|  | Normoblast | 31994 | 2003 | 50 | 28.61691756 | 20.15496076 | 15.67398119 |
|  | Plasmacell | 262 | 31 | 4 | 0.234344952 | 0.311933991 | 1.253918495 |
|  | Promyelocyte | 1168 | 159 | 10 | 1.044713375 | 1.599919501 | 3.134796238 |
|  | Pronormoblast | 117 | 21 | 1 | 0.104650227 | 0.211310123 | 0.313479624 |
| **4** | Blast | 843 | 220 | 8 | 3.02226365 | 3.205128205 | 2.962962963 |
|  | Granulocyte | 10933 | 2910 | 112 | 39.1962141 | 42.3951049 | 41.48148148 |
|  | Lymphocyte | 4045 | 917 | 32 | 14.50184634 | 13.35955711 | 11.85185185 |
|  | Monocyte | 2952 | 764 | 28 | 10.58330047 | 11.13053613 | 10.37037037 |
|  | Myelocyte/Metamyelocyte | 2787 | 719 | 30 | 9.991754204 | 10.47494172 | 11.11111111 |
|  | Normoblast | 3984 | 1167 | 46 | 14.28315348 | 17.00174825 | 17.03703704 |
|  | Plasmacell | 180 | 51 | 4 | 0.645323199 | 0.743006993 | 1.481481481 |
|  | Promyelocyte | 2057 | 77 | 7 | 7.374610117 | 1.121794872 | 2.592592593 |
|  | Pronormoblast | 112 | 39 | 3 | 0.401534435 | 0.568181818 | 1.111111111 |
| **5** | Blast | 79 | 41 | 1 | 0.108971529 | 0.369502523 | 0.31152648 |
|  | Granulocyte | 21688 | 5597 | 180 | 29.9161333 | 50.44160058 | 56.07476636 |
|  | Lymphocyte | 20494 | 2030 | 43 | 28.26914588 | 18.29488104 | 13.39563863 |
|  | Monocyte | 388 | 237 | 11 | 0.535201942 | 2.135904831 | 3.426791277 |
|  | Myelocyte/Metamyelocyte | 3108 | 912 | 26 | 4.287133083 | 8.219178082 | 8.099688474 |
|  | Normoblast | 25916 | 1905 | 46 | 35.74817921 | 17.16834895 | 14.33021807 |
|  | Plasmacell | 94 | 28 | 3 | 0.129662326 | 0.252343187 | 0.934579439 |
|  | Promyelocyte | 688 | 329 | 11 | 0.949017877 | 2.965032444 | 3.426791277 |
|  | Pronormoblast | 41 | 17 | 0 | 0.056554844 | 0.153208363 | 0 |
| **6** | Blast | 324 | 95 | 11 | 0.779052153 | 1.104908118 | 2.644230769 |
|  | Granulocyte | 22122 | 5133 | 241 | 53.19194979 | 59.69993022 | 57.93269231 |
|  | Lymphocyte | 6388 | 703 | 26 | 15.35983072 | 8.176320074 | 6.25 |
|  | Monocyte | 2509 | 839 | 60 | 6.032845223 | 9.758083275 | 14.42307692 |
|  | Myelocyte/Metamyelocyte | 2803 | 596 | 31 | 6.739762918 | 6.931844615 | 7.451923077 |
|  | Normoblast | 6437 | 1116 | 40 | 15.47765034 | 12.97976274 | 9.615384615 |
|  | Plasmacell | 645 | 41 | 0 | 1.550890861 | 0.476855083 | 0 |
|  | Promyelocyte | 223 | 35 | 2 | 0.536199476 | 0.407071412 | 0.480769231 |
|  | Pronormoblast | 138 | 40 | 5 | 0.33181851 | 0.465224471 | 1.201923077 |
| **7** | Blast | 212 | 119 | 9 | 0.217708312 | 0.810737158 | 2.760736196 |
|  | Granulocyte | 14833 | 5695 | 160 | 15.23239335 | 38.79956397 | 49.0797546 |
|  | Lymphocyte | 29810 | 3628 | 38 | 30.61266405 | 24.71726393 | 11.65644172 |
|  | Monocyte | 364 | 188 | 14 | 0.373801064 | 1.280828451 | 4.294478528 |
|  | Myelocyte/Metamyelocyte | 2194 | 974 | 32 | 2.253075643 | 6.635781442 | 9.81595092 |
|  | Normoblast | 48847 | 3780 | 58 | 50.16225431 | 25.75282736 | 17.79141104 |
|  | Plasmacell | 262 | 76 | 3 | 0.269054612 | 0.517781714 | 0.920245399 |
|  | Promyelocyte | 750 | 178 | 9 | 0.7701945 | 1.212699278 | 2.760736196 |
|  | Pronormoblast | 106 | 40 | 3 | 0.108854156 | 0.272516692 | 0.920245399 |
| **8** | Blast | 1401 | 696 | 8 | 0.206729251 | 0.927455893 | 1.436265709 |
|  | Granulocyte | 140288 | 35834 | 275 | 20.70066608 | 47.75065295 | 49.37163375 |
|  | Lymphocyte | 99948 | 5540 | 33 | 14.74816216 | 7.382335696 | 5.92459605 |
|  | Monocyte | 2620 | 989 | 18 | 0.386602882 | 1.317893502 | 3.231597846 |
|  | Myelocyte/Metamyelocyte | 34947 | 13866 | 114 | 5.156721726 | 18.47716007 | 20.46678636 |
|  | Normoblast | 391457 | 15722 | 83 | 57.76274978 | 20.95037578 | 14.90125673 |
|  | Plasmacell | 2781 | 230 | 3 | 0.410359777 | 0.306486861 | 0.538599641 |
|  | Promyelocyte | 3514 | 1786 | 17 | 0.518520049 | 2.379937104 | 3.052064632 |
|  | Pronormoblast | 742 | 381 | 6 | 0.109488297 | 0.507702148 | 1.077199282 |
| **9** | Blast | 2344 | 935 | 50 | 1.236423866 | 3.192215773 | 4.582951421 |
|  | Granulocyte | 73105 | 9830 | 403 | 38.56176053 | 33.5609423 | 36.93858845 |
|  | Lymphocyte | 30430 | 3835 | 117 | 16.0513559 | 13.09320587 | 10.72410632 |
|  | Monocyte | 4000 | 1734 | 68 | 2.109938337 | 5.920109252 | 6.232813932 |
|  | Myelocyte/Metamyelocyte | 13668 | 3456 | 125 | 7.209659298 | 11.79924889 | 11.45737855 |
|  | Normoblast | 62657 | 8522 | 282 | 33.0506016 | 29.09525435 | 25.84784601 |
|  | Plasmacell | 1215 | 201 | 9 | 0.64089377 | 0.686241038 | 0.824931256 |
|  | Promyelocyte | 1222 | 411 | 17 | 0.644586162 | 1.403209286 | 1.558203483 |
|  | Pronormoblast | 938 | 366 | 20 | 0.49478054 | 1.249573233 | 1.833180568 |
| **10** | Blast | 373 | 162 | 7 | 0.239027485 | 0.353326063 | 0.977653631 |
|  | Granulocyte | 41224 | 15012 | 307 | 26.41734327 | 32.74154853 | 42.87709497 |
|  | Lymphocyte | 48103 | 9649 | 54 | 30.82557402 | 21.04471101 | 7.541899441 |
|  | Monocyte | 1036 | 439 | 13 | 0.663894033 | 0.957470011 | 1.815642458 |
|  | Myelocyte/Metamyelocyte | 8985 | 3770 | 89 | 5.757806843 | 8.222464558 | 12.4301676 |
|  | Normoblast | 52879 | 15667 | 205 | 33.88615114 | 34.17011996 | 28.63128492 |
|  | Plasmacell | 1119 | 421 | 15 | 0.717082455 | 0.918211559 | 2.094972067 |
|  | Promyelocyte | 1968 | 612 | 20 | 1.261142333 | 1.33478735 | 2.793296089 |
|  | Pronormoblast | 362 | 118 | 6 | 0.231978417 | 0.25736096 | 0.837988827 |
| **11** | Blast | 1375 | 433 | 20 | 0.981469849 | 1.278341993 | 1.93986421 |
|  | Granulocyte | 64404 | 15295 | 432 | 45.97133394 | 45.15529051 | 41.90106693 |
|  | Lymphocyte | 10936 | 2076 | 54 | 7.806075834 | 6.12895607 | 5.237633366 |
|  | Monocyte | 4630 | 1570 | 91 | 3.304876656 | 4.635096835 | 8.826382153 |
|  | Myelocyte/Metamyelocyte | 26017 | 6212 | 180 | 18.57083714 | 18.33963155 | 17.45877789 |
|  | Normoblast | 26869 | 6453 | 187 | 19.17899155 | 19.05113368 | 18.13773036 |
|  | Plasmacell | 756 | 196 | 3 | 0.539629968 | 0.578649032 | 0.290979631 |
|  | Promyelocyte | 4472 | 1374 | 53 | 3.192096848 | 4.056447803 | 5.140640155 |
|  | Pronormoblast | 637 | 263 | 11 | 0.454688214 | 0.776452527 | 1.066925315 |
| **12** | Blast | 338 | 81 | 3 | 0.221168003 | 0.268968952 | 0.230237913 |
|  | Granulocyte | 55606 | 12107 | 518 | 36.38540815 | 40.20255687 | 39.75441289 |
|  | Lymphocyte | 15900 | 2489 | 89 | 10.40405693 | 8.264984227 | 6.830391404 |
|  | Monocyte | 1521 | 239 | 3 | 0.995256012 | 0.79362444 | 0.230237913 |
|  | Myelocyte/Metamyelocyte | 11286 | 2835 | 170 | 7.384917389 | 9.413913332 | 13.04681504 |
|  | Normoblast | 60236 | 10866 | 450 | 39.41501718 | 36.08168687 | 34.53568688 |
|  | Plasmacell | 3948 | 447 | 13 | 2.583346965 | 1.484310144 | 0.997697621 |
|  | Promyelocyte | 3715 | 965 | 54 | 2.430884999 | 3.204383198 | 4.144282425 |
|  | Pronormoblast | 275 | 86 | 3 | 0.179944381 | 0.285571974 | 0.230237913 |
| **13** | Blast | 153 | 71 | 2 | 0.049463019 | 0.176331802 | 0.428265525 |
|  | Granulocyte | 34319 | 13623 | 199 | 11.0949108 | 33.83335403 | 42.6124197 |
|  | Lymphocyte | 46322 | 1892 | 8 | 14.97533315 | 4.698869986 | 1.713062099 |
|  | Monocyte | 1001 | 404 | 6 | 0.323610994 | 1.003352788 | 1.284796574 |
|  | Myelocyte/Metamyelocyte | 26761 | 9600 | 124 | 8.651502318 | 23.84204644 | 26.55246253 |
|  | Normoblast | 186000 | 10258 | 72 | 60.13151344 | 25.47622004 | 15.41755889 |
|  | Plasmacell | 7876 | 849 | 2 | 2.546213978 | 2.108530982 | 0.428265525 |
|  | Promyelocyte | 6561 | 3380 | 51 | 2.121090643 | 8.394387185 | 10.92077088 |
|  | Pronormoblast | 329 | 188 | 3 | 0.106361655 | 0.466906743 | 0.642398287 |
| **14** | Blast | 648 | 217 | 5 | 0.739481222 | 1.25782518 | 1.170960187 |
|  | Granulocyte | 46320 | 9243 | 222 | 52.85921327 | 53.57639694 | 51.99063232 |
|  | Lymphocyte | 9987 | 1516 | 28 | 11.39691198 | 8.78738697 | 6.557377049 |
|  | Monocyte | 4328 | 1370 | 50 | 4.939004211 | 7.941108277 | 11.70960187 |
|  | Myelocyte/Metamyelocyte | 11798 | 2470 | 59 | 13.46357941 | 14.31718062 | 13.81733021 |
|  | Normoblast | 10878 | 2191 | 48 | 12.41369866 | 12.69997681 | 11.2412178 |
|  | Plasmacell | 294 | 53 | 3 | 0.335505369 | 0.307210758 | 0.702576112 |
|  | Promyelocyte | 3086 | 114 | 6 | 3.521665202 | 0.660792952 | 1.405152225 |
|  | Pronormoblast | 290 | 78 | 6 | 0.33094067 | 0.452121493 | 1.405152225 |
| **15** | Blast | 673 | 194 | 9 | 0.488857251 | 0.544637844 | 1.171875 |
|  | Granulocyte | 69182 | 17892 | 400 | 50.25278206 | 50.23020775 | 52.08333333 |
|  | Lymphocyte | 19629 | 4505 | 46 | 14.25821542 | 12.64738911 | 5.989583333 |
|  | Monocyte | 2400 | 678 | 47 | 1.74332452 | 1.903425042 | 6.119791667 |
|  | Myelocyte/Metamyelocyte | 10946 | 2945 | 63 | 7.951012581 | 8.267827063 | 8.203125 |
|  | Normoblast | 32156 | 8611 | 178 | 23.35764303 | 24.174621 | 23.17708333 |
|  | Plasmacell | 1160 | 277 | 4 | 0.842606851 | 0.777653004 | 0.520833333 |
|  | Promyelocyte | 1197 | 386 | 13 | 0.869483104 | 1.083660865 | 1.692708333 |
|  | Pronormoblast | 325 | 132 | 8 | 0.236075195 | 0.370578327 | 1.041666667 |
| **16** | Blast | 861 | 283 | 7 | 0.671706415 | 1.152749491 | 1.088646967 |
|  | Granulocyte | 24937 | 6502 | 167 | 19.45452134 | 26.48472505 | 25.97200622 |
|  | Lymphocyte | 38380 | 3408 | 66 | 29.94203509 | 13.88187373 | 10.26438569 |
|  | Monocyte | 6192 | 2498 | 95 | 4.830669132 | 10.17515275 | 14.77449456 |
|  | Myelocyte/Metamyelocyte | 8672 | 2476 | 65 | 6.765433255 | 10.08553971 | 10.1088647 |
|  | Normoblast | 39100 | 8212 | 207 | 30.5037408 | 33.45010183 | 32.19284603 |
|  | Plasmacell | 7627 | 272 | 3 | 5.950179824 | 1.107942974 | 0.466562986 |
|  | Promyelocyte | 1308 | 460 | 17 | 1.020432045 | 1.873727088 | 2.643856921 |
|  | Pronormoblast | 1104 | 439 | 16 | 0.861282093 | 1.788187373 | 2.488335925 |
